# 9.4-T MRI monitoring of early MASH progression and therapeutic response in a prefibrotic mouse model

**DOI:** 10.1186/s41747-026-00719-w

**Published:** 2026-05-06

**Authors:** Yuanyuan Li, Yun Wu, Gengxin Wang, Yanmin Zheng, Haiyang Tong, Hongyi Yang, Jin Yu, Pan Shi, Yong Zheng, Li Zhou, Xin Li, Pei Lv, Changlin Tian

**Affiliations:** 1 Anhui Province Key Laboratory of High Field Magnetic Resonance Imaging, High Magnetic Field Laboratory, Hefei Institute of Physical Science, Chinese Academy of Sciences, Hefei, China; 2 Science Island Branch, Graduate School of University of Science and Technology of China, Hefei, China; 3 School of Life Sciences, Division of Life Sciences and Medicine, University of Science and Technology of China, Hefei, China; 4 Anhui Provincial Engineering Laboratory of Peptide Drugs, University of Science and Technology of China, Hefei, China; 5 Beijing Life Science Academy, Beijing, China; 6 School of Biomedical Engineering, Division of Life Sciences and Medicine, University of Science and Technology of China, Hefei, China; 7 Suzhou Institute for Advanced Research, University of Science and Technology of China, Suzhou, China; 8 The International Peace Maternity and Child Health Hospital, National Center for Translational Medicine, School of Chemistry and Chemical Engineering, Zhangjiang Institute for Advanced Studies, Shanghai Jiao Tong University, Shanghai, China

**Keywords:** Fatty liver, Longitudinal monitoring, Magnetic resonance imaging, MASH, Semaglutide

## Abstract

**Objective:**

Early intervention in metabolic dysfunction-associated steatohepatitis (MASH) is critical to halt disease progression. However, noninvasive tools for monitoring early-stage MASH and therapeutic efficacy in preclinical models remain limited, impeding preclinical drug development. This study establishes an integrated approach using multiparametric magnetic resonance imaging (MRI) at 9.4 T to dynamically track disease development and drug response in a prefibrotic MASH mouse model.

**Materials and methods:**

Mice were fed a high-fat and high-cholesterol diet (HFHCD) for 16 weeks to induce early MASH without fibrosis and treated with the anti-MASH drug semaglutide for 8 weeks after modeling. Longitudinal MRI assessments—including proton density fat fraction (PDFF), ^1^H magnetic resonance spectroscopy (MRS), T_2_ mapping, and diffusion-weighted imaging (DWI)—were performed every 4 weeks and correlated with histopathology.

**Results:**

Histology confirmed early MASH after 16 weeks, without fibrosis. All MRI parameters strongly correlated with histopathological scores. HFHCD feeding led to significant changes: PDFF, MRS-derived liver fat content (LFC), and T_2_ values increased by 6.8-, 5.2-, and 2.5-fold, respectively, while apparent diffusion coefficient (ADC) decreased by 30% (*p* < 0.001). T_2_ and ADC also correlated with MRS-quantified saturated fatty acids. Semaglutide treatment effectively reversed these changes: PDFF decreased by 73%, LFC by 62%, T_2_ by 46% (*p* < 0.001), and ADC increased 1.4-fold (*p* = 0.017) compared to the vehicle group.

**Conclusion:**

This work demonstrates multiparametric MRI as a powerful noninvasive platform for monitoring early MASH dynamics and treatment response. By enabling longitudinal assessment in a prefibrotic model, this approach accelerates translational research in MASH diagnosis and drug development.

**Relevance statement:**

The established multiparametric MRI evaluation system provides a valuable noninvasive monitoring platform for preclinical early-stage MASH research, demonstrating significant potential to accelerate the translational progress in MASH diagnosis and drug development.

**Key Points:**

- A novel prefibrotic MASH model was established to assess early-stage MASH progression.
- Multiparametric MRI at 9.4 T enables noninvasive, longitudinal monitoring of early MASH.
- Semaglutide-induced improvement in steatosis and inflammation can be monitored by multiparametric MRI.

**Graphical Abstract:**

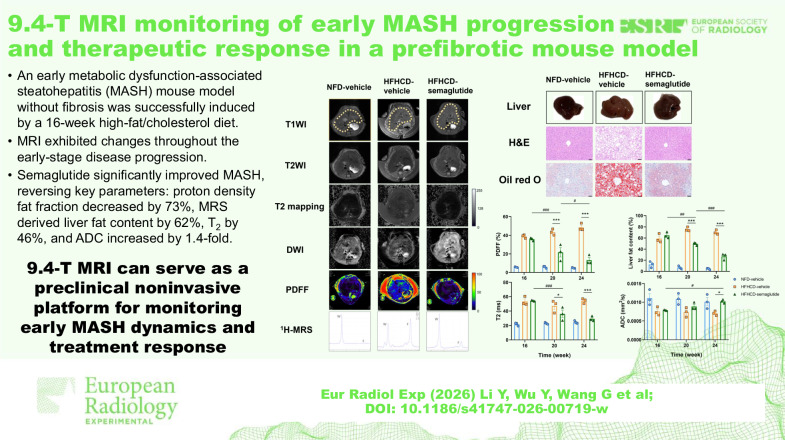

## Background

Metabolic dysfunction-associated steatotic liver disease (MASLD) affects more than 25% of the global population and is an increasingly prevalent chronic liver disease characterized by hepatic fat accumulation [1]. Approximately 20% of MASLD patients progress to metabolic dysfunction-associated steatohepatitis (MASH), a more severe form accompanied by hepatocyte ballooning and lobular inflammation that may further advance to irreversible cirrhosis, hepatocellular carcinoma or even liver failure [2]. Although early-stage MASH is reversible with timely intervention, its subtle clinical presentation necessitates dynamic monitoring to guide treatment [3].

The recent Food and Drug Administration approval of semaglutide—a glucagon-like peptide-1 (GLP-1) receptor agonist—for MASH highlights both the therapeutic potential of GLP-1-based agents and the substantial unmet need in early MASH management [4]. While numerous other candidates are under preclinical investigation, the lack of a reliable noninvasive modality for longitudinal drug efficacy assessment in murine models remains a major bottleneck in translational research.

Current treatment responses depend heavily on terminal histology, which fails to capture real-time *in vivo* dynamics [5, 6]. Conventional imaging techniques like ultrasound and computed tomography are limited by low sensitivity or radiation concerns [7–10], while elastography is costly and not widely accessible [11–13]. In contrast, MRI offers greater accessibility and, more importantly, is superior to magnetic resonance elastography in quantifying steatosis of early-stage MASH [14]. We hypothesize that ultra-high-field (9.4 T) multiparametric MRI can leverage its high signal-to-noise ratio to noninvasively detect subtle pathological changes in early MASH, enabling dynamic tracking of early-stage disease progression and treatment response.

While proton density fat fraction (PDFF) and MR spectroscopy (MRS) are widely applied for hepatic fat quantification in clinical settings, previous preclinical MRI studies have predominantly focused on late fibrotic stages [15–17]. Moreover, correlations between relaxation times and fibrosis remain controversial [18–20]. These gaps may arise from the use of established models, such as methionine-and choline-deficient diet or CCl_4_ models, which often advance rapidly to fibrosis and inadequately model early disease.

To address this, we developed a novel prefibrotic MASH mouse model using a 16-week high-fat high-cholesterol (HFHC) diet. Using longitudinal 9.4-T MRI, we quantified parameters including PDFF, liver fat fraction (LFC), T_2_, and apparent diffusion coefficient (ADC), and demonstrated their sensitivity to steatosis and inflammation progression. In semaglutide-treated MASH mice, multiparametric MRI—notably PDFF and T_2_ mapping—detected significant pathological and functional improvements. Our findings support the utility of 9.4-T multiparametric MRI as a sensitive, noninvasive tool for monitoring early MASH progression and therapy efficacy, providing a robust platform for accelerating preclinical drug development.

## Methods

### Animals and treatments

All animal-handling procedures were approved by the Ethics Committee of Hefei Institutes of Physical Science, Chinese Academy of Sciences. Male C57BL/6J mice (6–8 weeks of age, 18–20 g) were housed under standard conditions (*i.e*., group housing, 12 h:12 h light-dark cycle, room temperature 24 ± 2 °C) with *ad libitum* access to water and a HFHC diet (60 kJ% fat + 1% w ∙ w^-1^ cholesterol, *n* = 45) or a normal-fat diet (NFD) (*n* = 30). The modeling cycle lasted a total of 16 weeks. To noninvasively monitor longitudinal MRI changes within the same individuals during dietary intervention, we established two parallel mouse cohorts from the same source under identical conditions. One cohort underwent serial MRI every 4 weeks to track imaging parameter dynamics (NFD group, *n* = 3; HFHCD group, *n* = 9). The other cohort was sacrificed at corresponding time points for histological scoring (*n* = 3 per group and per time point), enabling a cross-sectional analysis correlating MRI variables with histology. During the modeling period, the body weight and status of the mice were regularly recorded weekly. After 16 weeks of modeling, the mice that responded well to the diet were divided into three treatment groups (*n* = 12 per group; in total 36 mice), which were balanced for body weight. GLP1R agonist semaglutide (self-synthesized) (30 nmol∙kg^-1^) or solvent vehicle (0.05% NaHCO_3_) was administered subcutaneously twice a week for a total of 8 weeks while the mice were maintained on the HFHC diet. The third group of animals was subcutaneously injected twice a week with vehicle while they were maintained on the NF diet. After 4 weeks and 8 weeks of administration, MRI scans and liver histopathology were performed (*n* = 3 per group). An oral glucose tolerance test was performed after 5 weeks of the intervention (*n* = 4–5 per group). At the end of the 8-week intervention period, blood biochemistry test was determined as described below (*n* = 3 per group).

### MRI image acquisition and data processing

*In vivo* MRI studies were conducted on a 9.4-T/400 mm scanner (Bruker BioSpin MRI GmbH) using a home-built radiofrequency coil except MRI-PDFF. MRI-PDFF experiments were performed in a horizontal 30 cm-inner-diameter 9.4-T magnet (uMR 9.4 T, United Imaging Healthcare) with a gradient insert having 1,000mT/m strength and 10,000 T ∙ m^-1^ ∙ s^-1^ slew rate. A 2-channel volume coil was used for transmitting and receiving. Mice were anesthetized with isoflurane (3.5% induction; 1.0–1.5% maintenance) in a 2:1 air/O_2_ ratio. Table 1 presents the sequence parameters for liver multiparametric MRI in detail.

**Table 1:** MRI acquisition parameters for liver multiparametric MRI

	Anatomical imaging	Quantitative imaging			
	T2WI	T2 map	DWI	^1^H-MRS	MRI-PDFF
Sequence	RARE	MSME	-	PRESS	PDFF
Water suppression	-	-	-	Yes	-
TE (ms)	16	8	17	16.5	1.04–5.79*
TR (ms)	2,000	2,500	2,500	2,500	45
b-value (s/mm^2^)	-	-	900	-	-
Averages	5	2	1	256	10
Flip angle (degree)	90	90	90	-	5
Echo spacing (ms)	8	8–128	-	-	-
Imaging size	192 × 192	192 × 192	128 × 128	-	134 × 168
Voxels size (mm^3^)	-	-	-	2 × 2 × 2	-
Field of view (mm^2^)	28 × 28	28 × 28	28 × 28	-	28 × 26
Bandwidth (kHz)	50	78	45	4	182.85
Slice					
Orientation	Axial	Axial	Axial	Axial	Axial
Number	15	15	15	-	15
Thickness (mm)	1	1	1	-	1
Gap (mm)	0	0	0	-	0
Distance (mm)	1	1	1	-	1

*DWI* Diffusion-weighted imaging, *MRI* Magnetic resonance imaging, *MSME* Multislice multiecho, ^*1*^*H-MRS* ^1^H-magnetic resonance spectroscopy, *PDFF* Proton density fat fraction, *PRESS* Point-resolved spectroscopy, *RARE* Rapid acquisition with relaxation enhancement, *T2WI* T2-weighted imaging, *TE* Echo time, *TR* Repetition time

* 1.04/1.99/2.94/3.89/4.84/5.79

An experienced researcher measured mean T_2_, ADC, and PDFF values by placing three regions of interest in both the left and right liver. All the MRS data were processed by the Bruker TopSpin software (3.1PV). In ^1^H-MR spectra, different peaks of fat can be resolved (Fig. S4). Peak assignments and frequencies are as follows: A-methyl (-CH_3_, around 0.9 ppm); B-methylene (-CH_2_, around 1.3 ppm); C-β-carboxyl (-CH_2_-CH_2_-COO, around 1.6 ppm); D-allylic (CH_2_-CH=CH-CH_2_, around 2.0 ppm); E-α-carbonyl (CH_2_-COO, around 2.2 ppm); F-diallylic (=CH-CH_2_-CH=, around 2.8 ppm); G-glycerol (-CH_2_-O-CO, around 4.1 ppm); H-glycerol (-CH_2_-O-CO, around 4.3 ppm); I-methine (CH=CH, around 5.3 ppm). Total percentage of fat in the liver was calculated as L / (L + W) × 100%, where W represents the amplitude of the water signal and L the total amplitude of all lipid signals. Based on the facts that all fatty acids contain three protons in position A and all unsaturated fatty acids contain four protons in position D, ƒ_UFA_ can be calculated as ƒ_UFA_ = 3/4*A_D_∕A_A_, where A_X_ denotes the peak area of peak X in Fig. S4b. The fraction of saturated fatty acids is calculated as ƒ_SFA_ = 1 - ƒ_UFA_ [21, 22].

### Glucose tolerance test

After 5 weeks of intervention, mice were fasted overnight for 8 h. Fasted blood samples were collected directly, and fasting blood glucose levels were measured using a glucometer (Abbott, FreeStyle Optium Neo). Subsequently, mice received an oral administration of either D-glucose (2 g∙kg^-1^ in approx. 200 μL water) to assess oral glucose tolerance. Blood samples were collected from the tail vein at 5, 10, 15, 30, 60 and 120 min after oral glucose administration to measure the continuous blood glucose level. The glucose tolerance curve was plotted, and the area under the curve was calculated.

### Blood biochemistry test

Fasted blood was collected and left at room temperature for 1 h. The blood was centrifuged at 3,000 rpm for 15 min, and the supernatant was collected and measured immediately. Serum levels of alanine aminotransferase (ALT) and aspartate aminotransferase (AST) were measured using a fully automated biochemistry analyzer (Radiometer Life Technology, Shenzhen).

### Liver histology

Part of livers were fixed with 4% paraformaldehyde, embedded in paraffin, cross-sectioned (4 μm) and stained for Hematoxylin-eosin (H&E) and Sirius Scarlet staining. In addition, fixed samples were dehydrated in 30% sucrose, cross-sectioned (10 μm) and stained with Oil red O. After staining, images were obtained by scanning through a slice scanner. Histologic scoring of H&E sections (steatosis score, fibrosis degree and NAFLD activity score (NAS)) was performed with reference to MASH diagnostic criteria and diagnostic algorithms for MASH described in the literature [23, 24] (Table S1).

### Data statistics

Data are presented as mean ± SEM; mice and individual data points were excluded only for technical reasons. All data were analyzed using GraphPad Prism version 8.0.1. Pearson correlation and linear regression analyses were performed to examine the relationships between MR parameters and histological findings, as well as modeling time. One-way ANOVA test was used to evaluate the difference among three or more groups. Two-way ANOVA was used to determine the effects of different time points or groups on MR parameters. Researchers were blinded only for the analysis of liver histology. *p*-values less than 0.05 were considered statistically significant.

## Results

### Construct HFHC diet-induced early-stage MASH mouse model

**Fig. 1:**
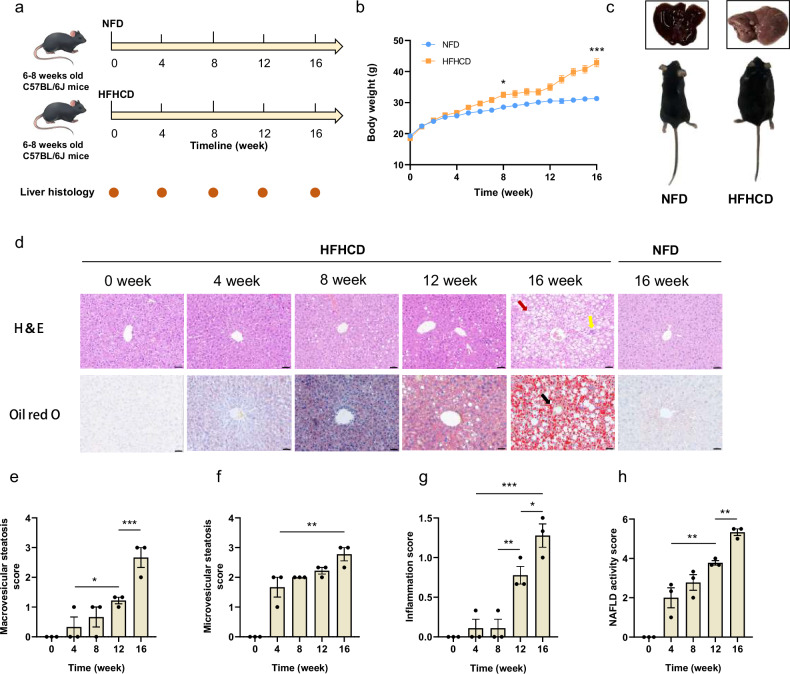
Establishment of MASH model in mice induced by HFHC diet. **a** Experimental schedule for constructing a high-fat high-cholesterol diet-induced MASH mouse model. **b** Body weight changes in mice fed with HFHC diet or NF diet. Data are presented as means ± S.E.M. (*n* = 12, * *p* < 0.05, *** *p* < 0.001). **c** Macroscopic pictures of representative livers and mice fed with HFHC diet or NF diet for 16 weeks. **d** Representative histological images of sectioned liver tissues in the HFHCD group and NFD group stained with H&E and Oil red O. Red arrowheads indicate steatosis, yellow arrowheads indicate hepatic lobular inflammation, and black arrowheads indicate lipid droplets. Scale bar, 50 μm. **e**–**h** Quantitative evaluation of macrovesicular steatosis score (**e**), microvesicular steatosis score (**f**), inflammation score (**g**), and NAS (**h**) from H&E-stained cross-sections. Data are presented as means ± S.E.M. (*n* = 3 per group and per time point, * *p* < 0.05, ** *p* < 0.01, *** *p* < 0.001). Statistical significance was assessed by one-way ANOVA followed by Tukey’s multiple comparisons test. HFHCD, High-fat high-cholesterol diet; H&E, Hematoxylin-eosin; MASH, Metabolic dysfunction-associated steatohepatitis; NFD, Normal-fat diet; S.E.M., Standard error of the mean

To simulate early-stage MASH, we established a high-fat, high-cholesterol diet (HFHCD)-induced MASH mouse model that closely mirrors human disease pathogenesis and allows for prolonged initial pathological progression. HFHCD-fed mice received a high-fat, high-cholesterol diet (60% fat with 1% cholesterol added), while the normal-fat diet (NFD) group was maintained on standard chow for 16 weeks (Fig. 1a). Weekly body weight measurements revealed a steady increase in both groups, with significantly greater weight gain in HFHCD-fed mice (Fig. 1b). Macroscopic examination showed that livers from the NFD group appeared healthy, reddish, and smooth, whereas HFHCD group livers were pale and granular, as a feature of a fatty liver (Fig. 1c).

Histological assessments performed at 4-week intervals demonstrated that HFHCD-fed mice developed progressive hepatic steatosis (red arrowheads), lobular inflammation (yellow arrowheads), and lipid droplet accumulation (black arrowheads), as visualized by H&E and Oil Red O staining, whereas NFD group livers showed no obvious pathological changes (Figs. 1d and S1). Notably, Sirius Red staining revealed no detectable collagen deposition in either group after 16 weeks (Fig. S2), confirming the absence of fibrosis and supporting the characterization of this model as early-stage MASH.

Histopathological scoring—including steatosis, inflammation, and NAS —was performed according to established MASH criteria [23–25]. Steatosis scores increased progressively from baseline, whereas lobular inflammation scores rose significantly after 8 weeks (Fig. 1e–h), indicating that steatosis predominated in the initial phase, with inflammation becoming prominent between weeks 12–16. NAS increased consistently throughout the study, and all HFHCD-fed mice met the diagnostic threshold for MASH (NAS ≥ 5) by week 16, confirming the successful establishment of an early-stage MASH model without fibrosis.

### Correlation between MRI parameters and liver pathological changes in HFHCD-induced MASH mice

**Fig. 2:**
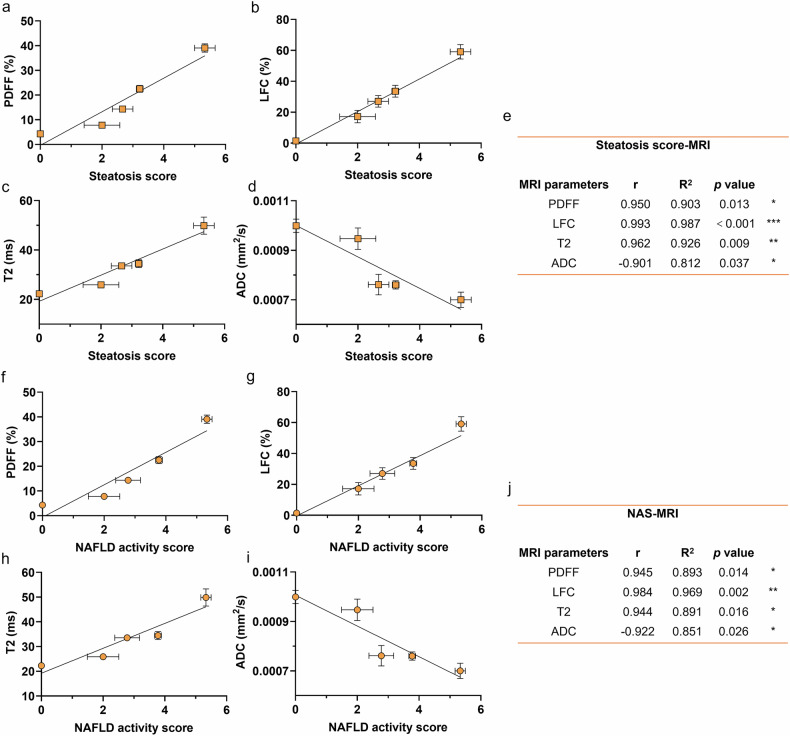
Association of MRI parameters and liver pathological changes over the course of MASH progression in HFHC diet-induced MASH mice. MRI parameters were obtained from the same cohort of mice through serial monitoring, while histological scores were from separate cohorts sacrificed every 4 weeks. **a**–**d** Linear regression and correlation analysis of PDFF (**a**), LCF (**b**), T_2_ (**c**) and ADC (**d**) with steatosis score during MASH progression in model mice, respectively. **e** The r, R^2^, and *p*-values of the correlation analyses between each MRI parameter and steatosis score. **f**–**i** Linear regression and correlation analysis of PDFF (**f**), LCF (**g**), T_2_ (**h**) and ADC (**i**) with steatosis score during MASH progression in model mice, respectively. **j** The r, R^2^, and *p*-values of the correlation analyses between each MRI parameter and NAS. Data are presented as means ± S.E.M. (steatosis score, *n* = 3; MRI scan, *n* = 9, * *p* < 0.05, ** *p* < 0.01, *** *p* < 0.001). Statistical significance was assessed by Pearson correlation analysis. ^1^H-MRS, ^1^H-magnetic resonance spectroscopy; ADC, Apparent diffusion coefficient; HFHC, High-fat high-cholesterol; LFC, Liver fat content; MASH, Metabolic dysfunction-associated steatohepatitis; MRI, Magnetic resonance imaging; NFD, Normal-fat diet; PDFF, Proton density fat fraction; S.E.M., Standard error of the mean

In this study, mouse livers were longitudinally monitored using 9.4-T MRI at 4-week intervals during HFHC diet feeding. Multiparametric sequences—including T_1_WI, T_2_WI, MRI-PDFF, ^1^H-MRS, T_2_ mapping, and DWI—were acquired to noninvasively assess hepatic changes. To evaluate the association between imaging parameters and pathological progression, linear regression analysis was performed between MRI-derived metrics and histopathological scores, including steatosis score (Fig. 2a–e) and the NAS, which incorporates both steatosis and inflammation (Fig. 2f–j).

PDFF, MRS-derived LFC, T_2_, and ADC values all exhibited significant correlations with both steatosis score and NAS. However, the strength of these correlations varied across parameters and scoring systems. LFC demonstrated a stronger correlation with steatosis score than with NAS, whereas PDFF showed comparable correlation strengths with both scores. Notably, LFC exhibited a stronger positive correlation with both pathological scores than PDFF (Fig. 2a, b).

T_2_ values also showed positive correlations with steatosis and NAS, whereas ADC was negatively correlated. Furthermore, T_2_ correlated more strongly with steatosis, while ADC showed a tighter inverse relationship with NAS. These differential associations highlight the complementary roles of multiparametric MRI in capturing distinct pathological features of early MASH.

### Longitudinal monitoring of MASH progression in HFHCD-induced MASH mice using MRI parameters

**Fig. 3:**
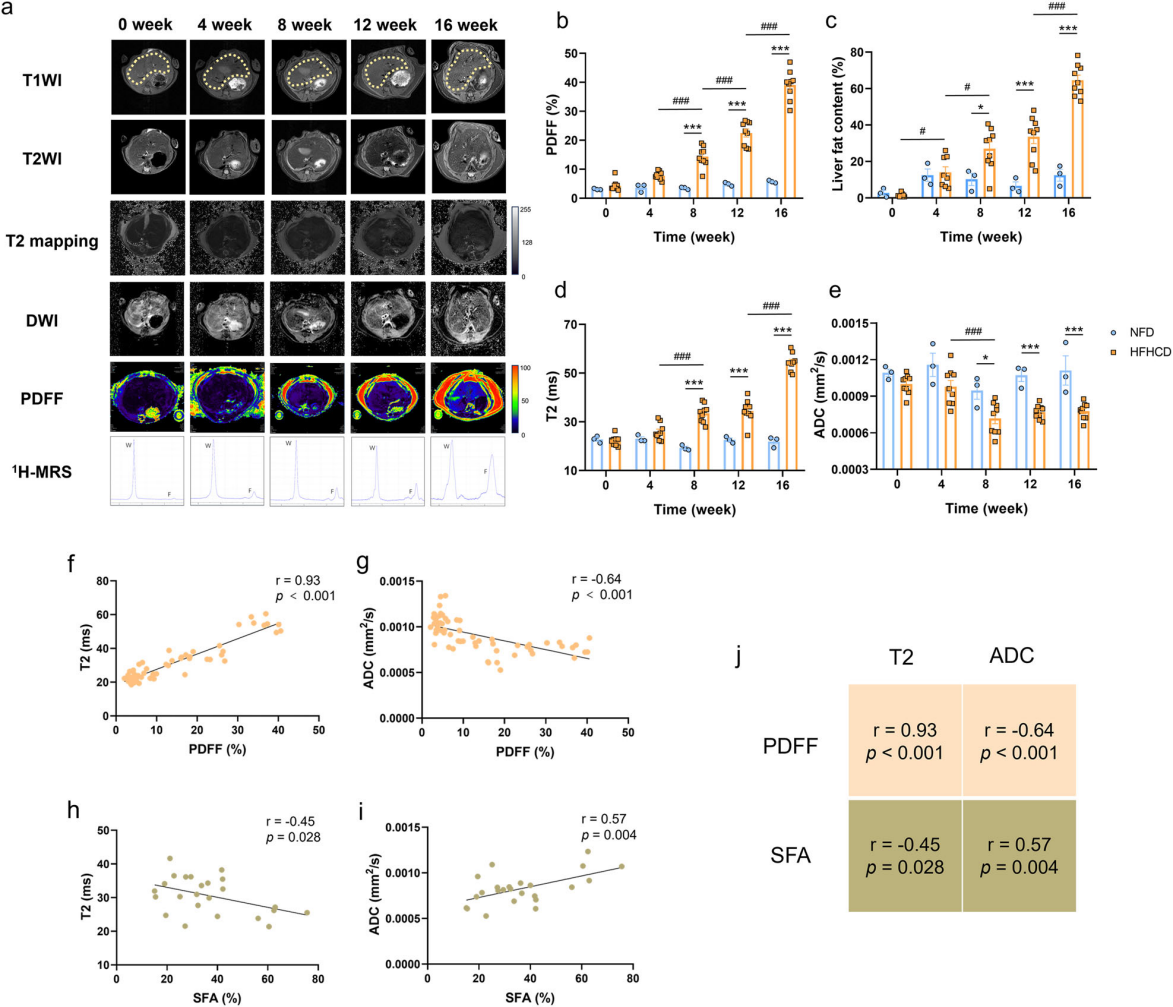
Longitudinal monitoring of MASH progression in HFHC diet-induced MASH mice based on 9.4-T MRI. **a** Representative MR images of livers during MASH progression. Yellow dotted lines indicate liver. All images were acquired using a 9.4-T MRI. **b**–**e** Quantitative analysis of conventional MRI, PDFF (**b**), MRS (**c**), T_2_ mapping (**d**) and DWI (**e**), over the course of MASH progression. Data are presented as means ± S.E.M. (*n* = 3). Statistical significance was assessed by two-way ANOVA followed by Tukey’s multiple comparisons test (within-group) and Sidak’s multiple comparisons test (between-group). Asterisks (*) denote significant differences compared to the NFD control group at the same time point (* *p* < 0.05, ** *p* < 0.01, *** *p* < 0.001). Number signs (^#^) indicate significant within-group differences at different time points (^#^ *p* < 0.05, ^###^ *p* < 0.001). **f**, **g** Correlation analysis of T_2_ values (**f**) and ADC values (**g**) with PDFF values during MASH progression in model mice, respectively. **h**, **i** Correlation analysis of T_2_ values (**h**) and ADC (**i**) values with SFA during MASH progression in model mice, respectively. **j** Correlation matrix for the analyses shown in **f**–**i**. ^1^H-MRS, ^1^H-magnetic resonance spectroscopy; ADC, Apparent diffusion coefficient; DWI, Diffusion-weighted imaging; HFHC, High-fat high-cholesterol; HFHCD, High-fat high-cholesterol diet; LFC, Liver fat content; MASH, Metabolic dysfunction-associated steatohepatitis; MRI, Magnetic resonance imaging; NFD, Normal-fat diet; PDFF, Proton density fat fraction; SFA, Saturated fatty acid; S.E.M., Standard error of the mean; T_2_WI, T_2_-weighted imaging

Having established the HFHC diet-induced mouse model of early-stage MASH, we next investigated whether multiparametric MRI could be used to monitor MASH progression over time. As mentioned above, MR images of mice livers were acquired every 4 weeks during modeling, including anatomical imaging and quantitative imaging (Fig. 3a). MRI-PDFF and ^1^H-MRS revealed a progressive increase in hepatic fat during the modeling period, visualized as elevated signal in liver regions (yellow dotted area) and an expanding lipid peak (the location corresponding to the letter “F”) on spectra. Quantitative analysis confirmed continuous increases in PDFF and LFC in the HFHCD group, with no significant changes in NFD controls (Fig. 3b, c). Statistically significant elevations in PDFF and LFC were observed by 8 weeks (4.1-fold and 2.7-fold *versus* NFD controls, *p* < 0.001 and *p* = 0.018, respectively) and increased further to 6.8-fold and 5.2-fold by 16 weeks (both *p* < 0.001), which was also in accordance with the histopathological findings (Fig. 1d–f). In addition, T_2_ and ADC values also showed dynamic changes during modeling. From week 4 onward, T_2_ increased and ADC decreased time-dependently in HFHCD mice, with no significant variation in the NFD group (Fig. 3d, e). Compared to the NFD group, the HFHCD group exhibited significantly increased T_2_ (1.7-fold, *p* < 0.001) and decreased ADC (20%, *p* = 0.017) by week 8, corresponding to the early MASH stage dominated by steatosis. By week 16, when hepatic pathology featured steatosis with concomitant lobular inflammation, T_2_ increased further to 2.5-fold *versus* NFD (*p* < 0.001), while ADC decreased an additional 30% *versus* NFD (*p* < 0.001). Correlation analysis in the longitudinal cohort demonstrated a variety in the strength of association with modeling time among the different MRI parameters (Fig. S3). Specifically, PDFF and LFC showed a stronger correlation. In contrast to PDFF, MRS can not only assess total fat content but also differentiate fatty acid types based on proton spectral differences between unsaturated and saturated fatty acids (SFA). Multiple studies have shown that saturated fatty acid levels are associated with various metabolic diseases, including MASH and type 2 diabetes [26]. Therefore, we further analyzed changes in SFA content during the MASH model development based on MRS results (Fig. S4). Overall, SFA levels did not exhibit a significant change. However, a downward trend was detected in the initial stage of modeling (weeks 4–8), a result consistent with previous studies [27]. We further examined correlations of T_2_ and ADC with PDFF and SFA to better interpret their associations with fat deposition (Fig. 3f–j). T_2_ strongly positively correlated with PDFF (r = 0.93, *p* < 0.001), but negatively with SFA (r = -0.45, *p* = 0.028). Conversely, ADC negatively correlated with PDFF (r = -0.64, *p* < 0.001) and positively with SFA (r = 0.57, *p* = 0.004).

### Effects of semaglutide on steatosis, inflammation and liver function in HFHCD-induced MASH mice

**Fig. 4:**
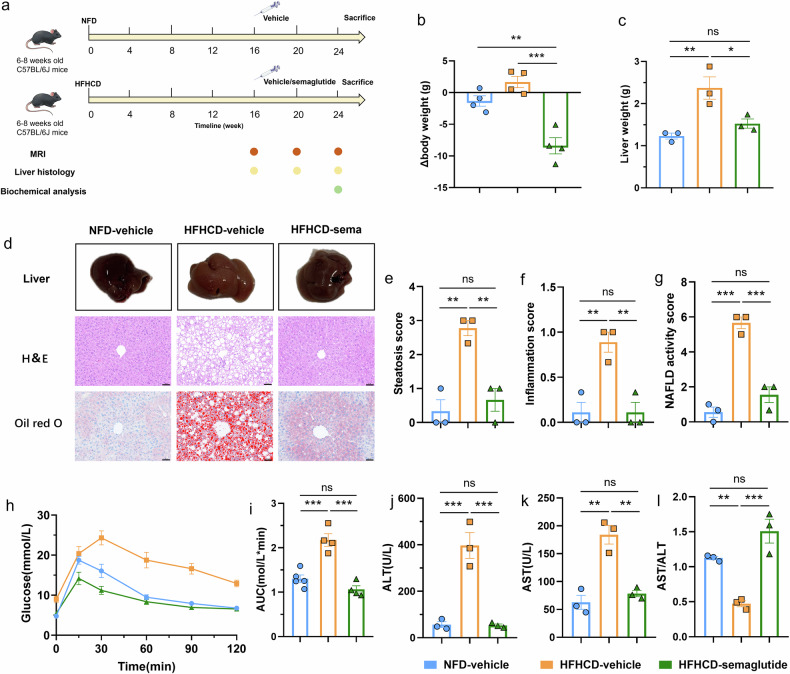
Semaglutide showed improvement in steatosis, inflammation and liver function in HFHC diet-induced MASH mice. **a** Experimental schedule for evaluating the therapeutic effect of a potential MASH drug. **b**, **c** Changes in body weight (*n* = 4) (**b**) and liver weight (*n* = 3) (**c**) of HFHC diet-induced MASH mice after 8 weeks of subcutaneous injection of semaglutide or vehicle. **d** Representative macroscopic pictures of livers and histological images of sectioned liver tissues stained with H&E and Oil red O after 8 weeks of intervention. Scale bar, 50 μm. **e**–**g** Quantitative evaluation of steatosis score (**e**), inflammation score (**f**), and NAS (**g**) from H&E-stained cross-sections (*n* = 3 per group and per time point). **h** Oral glucose tolerance tests performed in mice after 5 weeks of treatment with vehicle or semaglutide. **i** The area under the curve of the blood glucose change (*n* = 4–5). **j**–**l** Serum levels of ALT (**j**), AST (**k**) and AST/ALT (**l**) in mice after 8 weeks of treatment with vehicle or semaglutide (*n* = 3). Data are presented as means ± S.E.M. Statistical significance was assessed by one-way ANOVA followed by Tukey’s multiple comparisons test (* *p* < 0.05, ** *p* < 0.01, *** *p* < 0.001). ALT, Aminotransferase; AST, Aspartate aminotransferase; HFHC, High-fat high-cholesterol; HFHCD, High-fat high-cholesterol diet; H&E, Hematoxylin-eosin; MASH, Metabolic dysfunction-associated steatohepatitis; NAS, NAFLD activity score; NFD, Normal-fat diet; S.E.M., Standard error of the mean

Noninvasive monitoring of therapeutic response is essential for managing MASH patients and developing new drugs. To explore this, we treated established MASH mice with semaglutide and assessed improvements *via* liver histology. Mice previously fed an HFHCD for 16 weeks received twice-weekly subcutaneous semaglutide or vehicle for 8 weeks while maintaining the diet (Fig. 4a). After treatment, the semaglutide group showed significant reductions in body and liver weight, recovering to levels comparable to the NFD group (Fig. 4b, c). Macroscopically, livers in the semaglutide group appeared brighter red, in contrast to the pale livers of vehicle-treated mice. Histological evaluation at 4-week intervals revealed marked alleviation of hepatic steatosis and lobular inflammation in semaglutide-treated mice (Fig. 4d). This improvement was more pronounced after 8 weeks than at 4 weeks (Fig. S5), indicating progressive therapeutic benefit over time. Moreover, histological scores showed that after 8 weeks of treatment, NAS decreased significantly, inflammation was markedly alleviated, and the steatosis score also declined substantially. However, the steatosis score in the treatment group remained higher than that in the control group, indicating the persistence of mild residual hepatic steatosis in mice after the 8-week intervention. Oral glucose tolerance tests after 5 weeks of treatment demonstrated significantly enhanced glycemic control in semaglutide-treated mice, with a -63.8% reduction in AUC compared to vehicle (Fig. 4h, i). Furthermore, after 8 weeks of intervention, biochemical analysis showed substantial decreases in fasting serum levels of AST, ALT, and the AST/ALT ratio, confirming that semaglutide ameliorates liver injury in MASH mice (Fig. 4j–l).

### Utilizing multiparametric MRI for monitoring treatment response of semaglutide to MASH

**Fig. 5:**
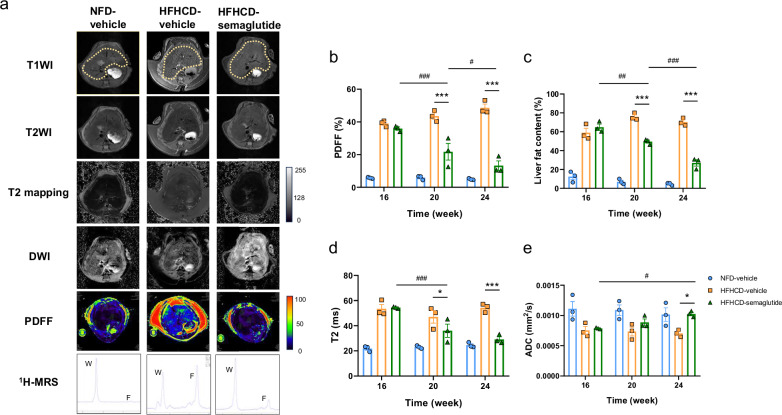
Monitoring the therapeutic response of MASH to semaglutide using conventional MRI. **a** Representative MR images of livers from vehicle- or semaglutide-treated groups at 8 weeks of intervention. Yellow dotted lines indicate liver. All images were acquired using a 9.4-T MRI. **b**–**e** Quantitative analysis of conventional MRI, PDFF (**b**), MRS (**c**), T_2_ mapping (**d**) and DWI (**e**), at weeks 4 and 8 of intervention with vehicle or semaglutide (*n* = 3 per group). Data are presented as means ± S.E.M. Statistical significance was assessed by two-way ANOVA followed by Tukey’s multiple comparisons test (within-group) and Sidak’s multiple comparisons test (between-group). Asterisks (*) denote significant differences compared to the HFHCD-vehicle control group at the same time point (* *p* < 0.05, ** *p* < 0.01, *** *p* < 0.001). Number signs (^#^) indicate significant within-group differences at different time points (^#^ *p* < 0.05, ^##^ *p* < 0.01, ^###^ *p* < 0.001).^1^H-MRS, ^1^H-magnetic resonance spectroscopy; ADC, Apparent diffusion coefficient; DWI, Diffusion-weighted imaging; HFHC, High-fat high-cholesterol; LFC, Liver fat content; MASH, Metabolic dysfunction-associated steatohepatitis; MRI, Magnetic resonance imaging; NFD, Normal-fat diet; PDFF, Proton density fat fraction; S.E.M., Standard error of the mean; T_2_WI, T_2_-weighted imaging

Having histologically confirmed the therapeutic effect of semaglutide on MASH, we further investigated whether multiparametric MRI could noninvasively monitor treatment response. Mice underwent MRI-PDFF, MRS, T_2_ mapping, and DWI at 4 and 8 weeks after semaglutide intervention (Fig. 5a). Consistent with clinical reports indicating that a decline in MRI-PDFF reflects histological improvement in MASH [28, 29], our model showed markedly reduced hepatic fat signal on MRI-PDFF (within yellow dotted regions) and a smaller lipid peak on ^1^H-MRS in semaglutide-treated mice compared to vehicle controls (Fig. 5a). Quantitatively, PDFF and LFC decreased by 50% and 35% at 4 weeks (both *p* < 0.001), and further declined by 73% and 62% at 8 weeks (both *p* < 0.001) relative to the HFHCD-vehicle group (Fig. 5b, c). We also evaluated T_2_ and ADC values as potential imaging biomarkers. After 8 weeks, semaglutide treatment significantly reduced T_2_ values by 46% *versus* the vehicle-treated HFHCD group (*p* < 0.001), while T_2_ values showed no significant difference *versus* the NFD group (Fig. 5d). Conversely, ADC values were significantly higher in the semaglutide group (1.4-fold *versus* vehicle-treated HFHCD, *p* = 0.017) but showed no difference compared to the NFD group (Fig. 5e). What’s more, the changes of T_2_ and ADC values after 4 and 8 weeks of semaglutide intervention were time-dependent and consistent with the above pathological changes (Figs. 4d and S5), providing MRI-based confirmation of MASH improvement. Notably, the trajectory of MRI parameter changes during treatment was opposite to that observed during MASH development (Fig. 3b–e), reinforcing their sensitivity to disease modulation.

## Discussion

Effective therapeutic intervention in the early stages of fatty liver hepatitis holds significant importance in reversing its pathological progression. However, to date, only two drugs—resmetirom and semaglutide—have recently received Food and Drug Administration conditional approval for treating adults with noncirrhotic MASH and moderate to severe fibrosis [4, 30]. Given that numerous other candidates remain in preclinical or early clinical trial phases (*e.g*., GLP-1 receptor agonists [31, 32], thyroid hormone receptor-β agonists [33] and fibroblast growth factor 21 analogs [34]), there is still a substantial unmet need for effective MASH therapies, especially in early-stage disease.

Preclinical murine models have played a pivotal role in elucidating the pathophysiological mechanisms underlying MASLD and MASH, as well as in evaluating the *in vivo* efficacy of therapeutic candidates. The establishment of a time-appropriate early-stage MASH murine model is important for dynamically monitoring disease progression and understanding initial pathological changes, which is substantial for laying the preclinical groundwork for early intervention. Additionally, current preclinical efficacy assessments predominantly rely on liver histopathology, which demands substantial numbers of animals and time while hindering longitudinal monitoring of treatment effects. Developing noninvasive, highly sensitive methods for dynamically tracking disease progression and drug efficacy in early MASH animal models holds significant practical value for accelerating preclinical drug development. Compared to ultrasound, computed tomography, and MRE, ultra-high-field MRI demonstrates a superior ability to detect minute steatosis, positioning it as a valuable tool for the monitoring of early MASH. While previous studies have investigated MRI parameter changes in animal models of liver fibrosis, these models (*e.g*., methionine-and choline-deficient diet or CCl_4_) are typically characterized by rapid pathological progression and advanced fibrosis severity, thereby failing to recapitulate or focus on early-stage pathological alterations. To bridge the gap, we combined ultra-high-field MRI with a 16-week HFHCD-fed early MASH mouse model, exploring its feasibility for early diagnosis and the dynamic monitoring of pharmacological intervention.

Pathological assessment at 4-week intervals revealed a progressive exacerbation of hepatic steatosis and lobular inflammation, effectively mimicking human early MASH pathogenesis. Critically, safranin staining confirmed the absence of fibrosis, verifying the successful establishment of a prefibrotic MASH model after 16 weeks of HFHCD. Thus, this model offers an ample window and pathological stage to study the correlation between MRI parameter dynamics and early disease progression.

Using ultra-high-field MRI, we longitudinally monitored MASH progression in HFHCD-fed mice through multiparametric imaging, including structural MRI, T_2_ mapping, DWI, ^1^H-MRS, and MRI-PDFF. Cross-sectionally, MRI parameters (PDFF, LFC, T_2_, ADC) exhibited significant and differential correlations with steatosis scores and NAS, indicating that different MRI parameters correlate not only with steatosis but also with hepatic lobular inflammation in early MASH mice. Longitudinally, PDFF and LFC showed the most substantial changes during early MASH (Fig. 3b, c), aligning with histopathological fat deposition. Notably, studies indicate that hepatic saturated fatty acid content correlates with diseases such as MASH and type 2 diabetes. ^1^H-MRS findings revealed altered saturated fatty acid levels during early MASH development in mice, suggesting MRS can reflect metabolic changes in early-stage MASH. From week 8, T_2_ values gradually increased while ADC values remained significantly lower than in the NFD group. This reflects distinct patterns of intracellular and extracellular water content changes, along with characteristic alterations in collagen fiber deposition within the interstitial space, which become pronounced during early MASH development [35, 36]. Among all parameters, PDFF and T_2_ demonstrated the most sensitivity to early disease, showing significant increases from week 8 (Fig. 3b, d). Moreover, within the longitudinal cohort, the strength of correlation between MRI parameters and modeling time was found to vary (Fig. S3). Specifically, PDFF and LFC showed a stronger correlation. This may be partially attributable to the inherent sensitivity of different MRI parameters to distinct pathological changes during HFHC diet-induced MASH progression, and the temporal dynamics of these pathological changes themselves also vary (Fig. 1e–h). Correlation analyses further revealed an inverse relationship between T_2_ and SFA, and a positive correlation between ADC and SFA. These results underscore the complementary role of multiparametric MRI in capturing early metabolic and structural alterations in MASH, providing novel insights into lipid dynamics at the molecular level. Overall, multiparametric MRI is noninvasively feasible to monitor early-stage MASH progression in HFHCD-fed mice at 9.4 T, providing a valuable tool for staging and refining early drug intervention.

Our study further demonstrates the value of multiparametric MRI for the noninvasive and dynamic monitoring of therapeutic efficacy in MASH mice treated with semaglutide. Compared to the vehicle-treated group, semaglutide treatment significantly reduced body weight and liver weight, improved hepatic steatosis and lobular inflammation, and restored liver function, which aligns with previous findings on the weight loss, anti-inflammatory effects, and insulin sensitivity improvement associated with GLP-1 receptor agonists [37–39]. Longitudinal MRI monitoring revealed a significant decrease in hepatic PDFF and LFC, consistent with the trend of histological improvement in steatosis. Furthermore, changes in T_2_ and ADC values after treatment—a decrease in T_2_ and an increase in ADC—were time-dependent and exhibited a trend opposite to that observed during MASH progression.

Notably, after 8 weeks of treatment, T_2_ and ADC values returned to levels comparable to those in the NFD-vehicle group, whereas PDFF and LFC remained elevated relative to controls (Fig. 5b–e). This finding is consistent with the histological scores (Fig. 4e–g) and aligns with prior clinical reports suggesting that MRI-PDFF and MRS can reflect improvements in MASH-related steatosis [28, 29, 40]. Compared to the response of steatosis to semaglutide, T_2_ and ADC values may be more sensitive to the resolution of ballooning degeneration and inflammation. Collectively, our findings showed that multiparametric MRI can comprehensively monitor the temporal dynamics of semaglutide treatment response in a preclinical MASH model, extending beyond mere fat quantification and supporting their utility in accelerating the development of multiple MASH candidates in the preclinical stage.

This study acknowledges several experimental and instrumental limitations. First, the model utilized in our study was a prefibrotic MASH model induced in male C57BL/6J mice by a 16-week high-fat high-cholesterol diet. It is important to note that extended dietary induction, different genetic backgrounds, or female sex could lead to different findings, such as insights into fibrotic transition dynamics with fibrotic models. Second, technical constraints in multiparametric MRI acquisition (*e.g*., motion artifacts in abdominal imaging, magnetic field uniformity and RF field inhomogeneity at high field) may influence quantitative accuracy. Third, validation across diverse liver disease models, including high-fat diet, methionine-choline-deficient, and genetically modified strains, is essential to establish broader applicability, which will be an area of future exploration. Despite these limitations, we have achieved substantial success in longitudinal monitoring of early-stage MASH progression using ultra-high-field 9.4-T MRI in HFHCD-induced MASH mouse model, which has superior pathophysiological similarities to human early MASH progression.

In summary, we successfully combined ultra-high-field multiparametric MRI with a prefibrosis MASH mouse model to conduct a longitudinal monitor of early MASH pathological progression in HFHCD mice, and tracked the response to anti-MASH semaglutide therapy, delivering a transformative noninvasive modality to redefine early-stage MASH diagnosis and accelerate preclinical therapeutic development.

## Supplementary information

**Additional file 1:** **Figure S1.** Representative histological images of sectioned liver tissues in NFD group stained with H&E and Oil red O during modeling. Scale bar, 50 μm. HFHCD, high-fat high-cholesterol diet; H&E, hematoxylin-eosin; NFD, normal-fat diet. **Figure S2.** Representative histological images of sectioned liver tissues stained with sirius scarlet during modeling. Scale bar, 50 μm. HFHCD, high-fat high-cholesterol diet; NFD, normal-fat diet. **Figure S3.** Association of MRI parameters and modeling time in HFHC diet-induced MASH mice. (a-d) Correlation analysis of PDFF (a), LFC (b), T2 (c) and ADC (d) with modeling time in HFHC diet-induced MASH mice, respectively. (e) The r, R2, and *p*-values of the correlation analyses between each MRI parameter and time. HFHC, high-fat high-cholesterol; LFC, liver fat content; MASH, metabolic dysfunction-associated steatohepatitis; MRI, magnetic resonance imaging; PDFF, proton density fat fraction. **Figure S4.** MR spectrum and SFA content in livers of HFHC diet-induced MASH mice (a) Schematic illustration of a triglyceride molecule. Triglyceride are composed of glycerol and three fatty acids. Hydrogen atoms are represented by light gray circles, carbon atoms by dark gray spheres, and oxygen atoms by red spheres. The letters in the circles indicate hydrogen atoms in distinct chemical environments, corresponding respectively to the peaks in (b). (b) MR spectrum of livers in HFHC diet-induced MASH mice. A-methyl (-C**H3**, around 0.9 ppm); B-methylene (-C**H2**, around 1.3 ppm); C-β-carboxyl (-C**H2**-CH2-COO, around 1.6 ppm); D-allylic (CH3-CH=CH-C**H2**, around 2.0 ppm); E-α- carbonyl (C**H2**-COO, around 2.2 ppm); F-diallylic (=CH-C**H2**-CH=, around 2.8 ppm); G-glycerol (-C**H2**-O-CO, around 4.1 ppm); H- glycerol (-C**H2**-O-CO, around 4.3 ppm); I-methine (C**H**=C**H**, around 5.3 ppm). (c) The content of SFA in the livers of HFHCD group during the modeling. Data are presented as means ± S.E.M. (n = 6). MRS, magnetic resonance spectroscopy; HFHC, high-fat high-cholesterol; MASH, metabolic dysfunction-associated steatohepatitis; SFA, saturated fatty acid; S.E.M., standard error of the mean. **Figure S5.** Representative histological images of sectioned liver tissues stained with H&E and Oil red O after 4 weeks of intervention. Scale bar, 50 μm. HFHCD, high-fat high-cholesterol diet; H&E, hematoxylin-eosin; NFD, normal-fat diet. **Table S1.** NASH histological scoring system.

## Data Availability

All data generated or analyzed during this study are included in this published article and its supplementary information files.

## Abbreviations

^1^H-MRS: ^1^H-magnetic resonance spectroscopy
ADC: Apparent diffusion coefficient
ALT: Aminotransferase
AST: Aspartate aminotransferase
DWI: Diffusion-weighted imaging
GLP-1: Glucagon-like peptide-1
H&E: Hematoxylin-eosin
HFHC: High-fat high-cholesterol
HFHCD: High-fat high-cholesterol diet
LFC: Liver fat content
MASH: Metabolic dysfunction-associated steatohepatitis
MASLD: Metabolic dysfunction-associated steatotic liver disease
MRI: Magnetic resonance imaging
NAFLD: Non-alcoholic fatty liver disease
NAS: NAFLD activity score
NFD: Normal-fat diet
PDFF: Proton density fat fraction
S.E.M.: Standard error of the mean
SFA: Saturated fatty acid
T_2_WI: T_2_-weighted imaging
