# Supplementary material for: 9.4-T MRI monitoring of early MASH progression and therapeutic response in a prefibrotic mouse model
Source: Eur Radiol Exp. 2026 May 6;10:59. doi: 10.1186/s41747-026-00719-w (PMC13149678; doi:10.1186/s41747-026-00719-w)
Supplement: Supplementary file 1 — Additional file 1: Figure S1. Representative histological images of sectioned liver tissues in NFD group stained with H&E and Oil red O during modeling. Scale bar, 50 μm. HFHCD, high-fat high-cholesterol diet; H&E, hematoxylin-eosin; NFD, normal-fat diet. Figure S2. Representative histological images of sectioned liver tissues stained with sirius scarlet during modeling. Scale bar, 50 μm. HFHCD, high-fat high-cholesterol diet; NFD, normal-fat diet. Figure S3. Association of MRI parameters and modeling time in HFHC diet-induced MASH mice. (a-d) Correlation analysis of PDFF (a), LFC (b), T2 (c) and ADC (d) with modeling time in HFHC diet-induced MASH mice, respectively. (e) The r, R2, and p-values of the correlation analyses between each MRI parameter and time. HFHC, high-fat high-cholesterol; LFC, liver fat content; MASH, metabolic dysfunction-associated steatohepatitis; MRI, magnetic resonance imaging; PDFF, proton density fat fraction. Figure S4. MR spectrum and SFA content in livers of HFHC diet-induced MASH mice (a) Schematic illustration of a triglyceride molecule. Triglyceride are composed of glycerol and three fatty acids. Hydrogen atoms are represented by light gray circles, carbon atoms by dark gray spheres, and oxygen atoms by red spheres. The letters in the circles indicate hydrogen atoms in distinct chemical environments, corresponding respectively to the peaks in (b). (b) MR spectrum of livers in HFHC diet-induced MASH mice. A-methyl (-CH3, around 0.9 ppm); B-methylene (-CH2, around 1.3 ppm); C-β-carboxyl (-CH2-CH2-COO, around 1.6 ppm); D-allylic (CH3-CH=CH-CH2, around 2.0 ppm); E-α- carbonyl (CH2-COO, around 2.2 ppm); F-diallylic (=CH-CH2-CH=, around 2.8 ppm); G-glycerol (-CH2-O-CO, around 4.1 ppm); H- glycerol (-CH2-O-CO, around 4.3 ppm); I-methine (CH=CH, around 5.3 ppm). (c) The content of SFA in the livers of HFHCD group during the modeling. Data are presented as means ± S.E.M. (n = 6). MRS, magnetic resonance spectroscopy; HFHC, high-fat high-chole [file 41747_2026_719_MOESM1_ESM.pdf]

9.4-T MRI monitoring of early MASH progression and therapeutic response in a prefibrotic mouse model

ELECTRONIC SUPPLEMENTARY MATERIAL

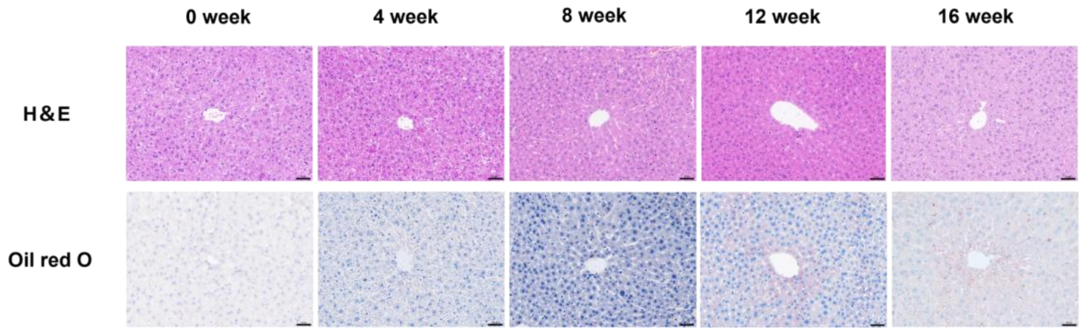

Figure S1. Representative histological images of sectioned liver tissues in NFD group stained with H&E and Oil red O during modeling. Scale bar, 50 μm. HFHCD, high-fat high-cholesterol diet; H&E, hematoxylin-eosin; NFD, normal fat diet.

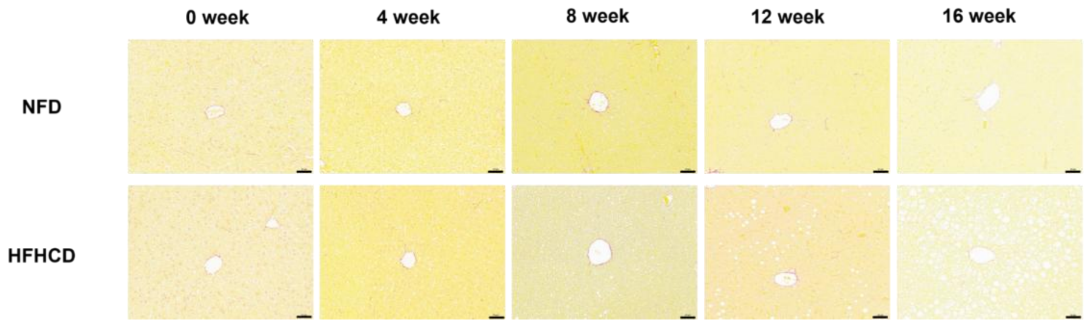

Figure S2. Representative histological images of sectioned liver tissues stained with sirius scarlet during modeling. Scale bar, 50 μm. HFHCD, high-fat high-cholesterol diet; NFD, normal fat diet.

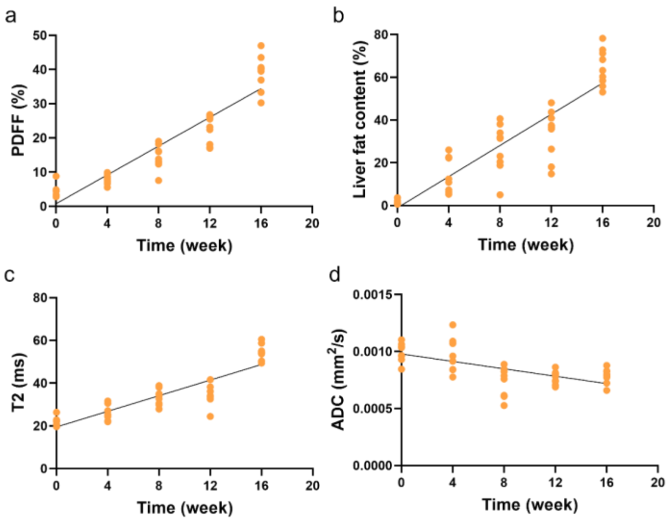

e

MRI-Time

MRI parameters	r	R^2	*p* value
**PDFF**	0.930	0.864	< 0.001
**LFC**	0.900	0.811	< 0.001
**T2**	0.886	0.785	< 0.001
**ADC**	-0.607	0.368	< 0.001

Figure S3. Association of MRI parameters and modeling time in HFHC diet-induced MASH mice. (a-d) Correlation analysis of PDFF (a), LFC (b), T_2 (c) and ADC (d) with modeling time in HFHC diet-induced MASH mice, respectively. (e) The r, R^2, and *p* values of the correlation analyses between each MRI parameter and time. HFHC, high-fat high-cholesterol; LFC, liver fat content; MASH, metabolic dysfunction-associated steatohepatitis; MRI, magnetic resonance imaging; PDFF, proton density fat fraction.

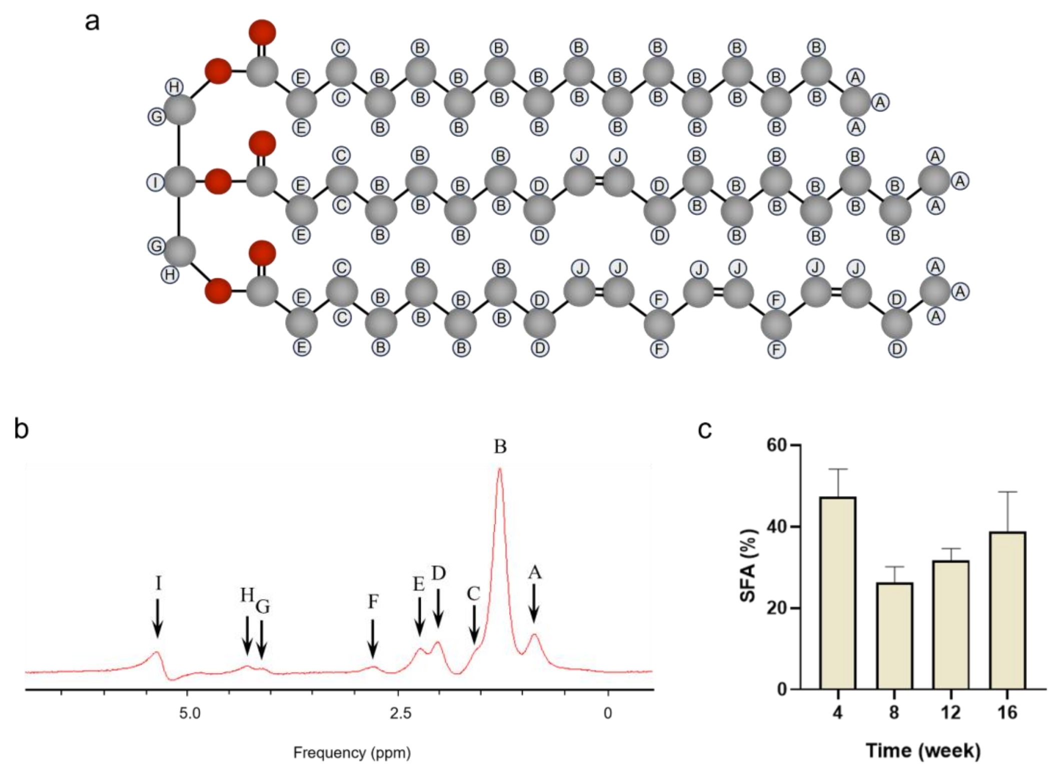

Figure S4. MR spectrum and SFA content in livers of HFHC diet-induced MASH mice (a) Schematic illustration of a triglyceride molecule. Triglyceride are composed of glycerol and three fatty acids. Hydrogen atoms are represented by light gray circles, carbon atoms by dark gray spheres, and oxygen atoms by red spheres. The letters in the circles indicate hydrogen atoms in distinct chemical environments, corresponding respectively to the peaks in (b). (b) MR spectrum of livers in HFHC diet-induced MASH mice. A-methyl ($-C\mathbf{H_3}$**,** around 0.9 ppm); B-methylene ($-C\mathbf{H_2}$, around 1.3 ppm); C-β-carboxyl ($-C\mathbf{H_2}-CH_2-COO$, around 1.6 ppm); D-allylic ($CH_3-CH=CH-C\mathbf{H_2}$, around 2.0 ppm); E-α- carbonyl ($C\mathbf{H_2}-COO$, around 2.2 ppm); F-diallylic ($=CH-C\mathbf{H_2}-CH=$, around 2.8 ppm); G-glycerol ($-C\mathbf{H_2}-O-CO$, around 4.1 ppm); H- glycerol ($-C\mathbf{H_2}-O-CO$, around 4.3 ppm); I-methine ($C\mathbf{H}=C\mathbf{H}$, around 5.3 ppm). (c) The content of SFA in the livers of HFHCD group during the modeling. Data are presented as means ± S.E.M. (n = 6). MRS, magnetic resonance spectroscopy; HFHC, high-fat high-cholesterol; MASH, metabolic dysfunction-associated steatohepatitis; SFA, saturated fatty acid; S.E.M., standard error of the mean.

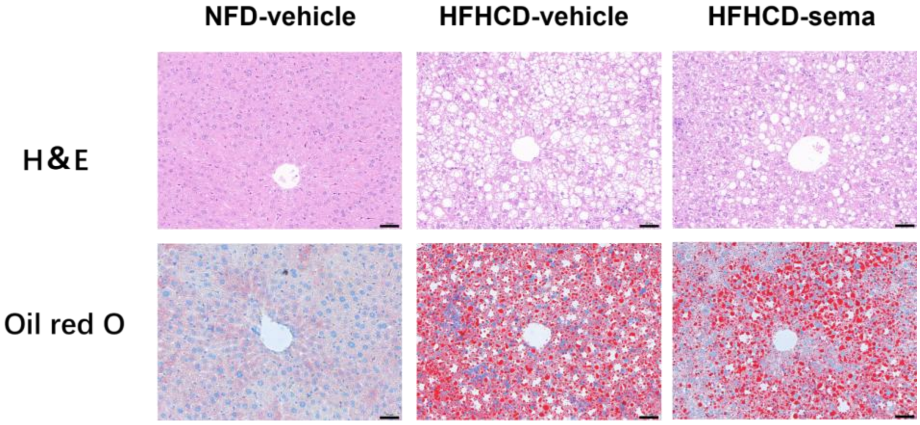

Figure S5. Representative histological images of sectioned liver tissues stained with H&E and Oil red O after 4 weeks of intervention. Scale bar, 50 μm. HFHCD, high-fat high-cholesterol diet; H&E, hematoxylin-eosin; NFD, normal fat diet.

Table S1. NASH histological scoring system

score	0	1	2	3
Steatosis	< 5%	5-33%	34-66%	> 66%
Lobular inflammation	No foci	< 2 foci/200 × field	2-4 foci/200 × field	> 4 foci/200 × field
Balloning of hepatocytes	none	Few ballooned	Many ballooned	

NAFLD activity score (0-8) = Steatosis (0-3) + Lobular inflammation (0-3) + Ballooning of hepatocytes (0-2)
